# Associations between Inter-Limb Asymmetries in Jump and Change of Direction Speed Tests and Physical Performance in Adolescent Female Soccer Players

**DOI:** 10.3390/ijerph18073474

**Published:** 2021-03-27

**Authors:** Elena Pardos-Mainer, Chris Bishop, Oliver Gonzalo-Skok, Hadi Nobari, Jorge Pérez-Gómez, Demetrio Lozano

**Affiliations:** 1 Health Sciences Faculty, Universidad San Jorge, Autov A23 km 299, 50830 Villanueva de Gállego, 50830 Zaragoza, Spain; epardos@usj.es; 2 Faculty of Science and Technology, London Sports Institute, London NW4 4BT, UK; C.Bishop@mdx.ac.uk; 3 Head of Return to Play, Sevilla Futbol Club, 41005 Sevilla, Spain; oligons@hotmail.com; 4 Department of Physical Education and Sports, University of Granada, 18010 Granada, Spain; hadi.nobari1@gmail.com; 5 HEME Research Group, Faculty of Sport Sciences, University of Extremadura, 10003 Cáceres, Spain; jorgepg100@gmail.com; 6 Department of Exercise Physiology, Faculty of Sport Sciences, University of Isfahan, Isfahan 81746-7344, Iran; 7 Sports Scientist, Sepahan Football Club, Isfahan 81887-78473, Iran

**Keywords:** athletic performance, youth sports, females, football

## Abstract

The association between asymmetries in jump and change of direction (COD) with physical performance in several sports show inconclusive results. The purposes of this study were to: (1) measure inter-limb asymmetries in three distinct groups in adolescent female soccer players and, (2) to determine the association between inter-limb asymmetries and physical performance in different age groups. Fifty-four players were distributed in three age groups: U-18, U-16 and U-14. All of them performed a series of jumps, sprints and change of direction speed tests. Asymmetries were assessed as the percentage difference between limbs, with the equation: 100/Max value (right and left) * in value (right and left) * −1 + 100. Mean inter-limb asymmetries were 2.91%, 4.82% and 11.6% for 180° COD, single leg hop and single leg countermovement jump tests respectively, but higher percentages of asymmetries were observed in many players individually. U-18 and U-16 showed significant differences on 180° left COD compared to U-14. Effect size (ES): 0.80 and 0.74, respectively; U-18 presented differences on single left leg hop test compared to U-14, ES: −0.72; U-16 also showed differences on 40 m speed compared to U-14, ES 0.87 (All *p* < 0.05). Jumping and COD physical tests show asymmetries in adolescent female soccer players, but these asymmetries do not interfere with physical performance. The largest asymmetry was observed in the single leg countermovement jump, and no asymmetries between groups were found. Due to the high variability in the direction of asymmetries, it is recommended to consider players’ individual asymmetries for designing specific training programs.

## 1. Introduction

Women’s football has witnessed a notable increase in popularity during the last decade [1], particularly at the youth level, where a ~4% increase in participation has been observed in the last 5 years [1]. Such impact has resulted in both increased demands during competition and training, as well as greater skill levels during matches [2]. Given these changes to the youth female game, a better understanding of the physical demands players face face across different age categories seems warranted.

In soccer, many high-intensity actions are performed unilaterally such as: jumping, sprinting, changing direction and kicking [3,4]. Given the prevalence of these actions occurring on one side, and the associated positional differences in soccer, inter-limb asymmetries should be expected in athletes who compete in this sport. Inter-limb asymmetries have been a reference on researches in latest years, it means to the concept of comparing the performance or function between limbs [5]. Literature has showed the requirement to investigate the connection between asymmetry and measures of physical performance [6,7,8], as the prevalence of asymmetry alone provides us with limited information as to the impact on athletic performance. Previous research in team sport athletes has shown that asymmetry may simply be a by-product of competing in a single sport over time [9]. Furthermore, the existing evidence base is still unclear as to whether asymmetry is consistently a problem for team sport athletes. 

Only three studies have investigated the relationship between inter-limb asymmetries and measures of physical performance in female soccer players [6,10,11]. Bishop et al. [10] showed jump height asymmetry of 9.2% in the unilateral drop jump, which showed significant associations with linear sprint (r = 0.52–0.58) and change of direction (COD) speed (r = 0.52–0.66) tests in adult female soccer players. Otherwise, no relationships were found between countermovement jump (CMJ) asymmetry and linear or COD speed. Similarly, Bishop et al. [6], found jump height asymmetry of 12.5% from the unilateral CMJ was associated with reduced speed performance (r = 0.49–0.59) in academy youth female soccer players. In contrast, Loturco et al. [11] showed jump height asymmetry of 9.8% and 10.6% from the unilateral squat jump and CMJ respectively, with no association with speed and power performance in professional female soccer players. Thus, with this conflicting evidence and lack of studies in female soccer players, it is not obvious if the differences in results are linked to the level (i.e., youth vs. professional), or motor activities performed (i.e., unilateral vs. bilateral horizontal or vertical jumps, straight running vs. COD). Thus, more researches are necessary to establish a correlation between inter-limb asymmetry and physical performance, specifically in adolescent female soccer players. 

Inter-limb asymmetries has also been associated with injury risk [12,13], highlighting the important to analyse the effect of exercise-induced fatigue during training or sport practice due to inter-limb asymmetries on the risk of injuries [12,14]. Some studies have observed that greater inter-limb asymmetries and lower physical fitness showed a higher predisposition to injury [15]. It is known that athletic performance is influenced by players fatigue and it could be accentuated by asymmetries [7,8,9,10,11,12,13,14,15,16]. A reduced athletic performance was found with only 5% differences inter-lib asymmetries [7], however, other studies did not find a relationship between asymmetries and deterioration on physical performance [17]. So the influence of asymmetries on fatigue and the negative effects on exercise performance required more investigations.

Furthermore, there is an insufficiency of literature researching how side to side differences interact with physical performance between different chronological age groups. Read et al. [18] and Kellis et al. [19] examined different chronological age groups in youth male soccer players. Read et al. [18] showed that single-leg countermovement jump landing force asymmetry was significantly higher for circa and post-peak height velocity (PHV) (*p* < 0.001; d = 0.41–0.43) compared with those who were pre-PHV; whereas Kellis et al. [19] founded that asymmetry, during diverse strength parameters using a isokinetic dynamometry, was not affected by age. On the other hand, Bishop et al. [7] recently observed elite male soccer players (under (U-16 to U-23) and they founded that jump height of single leg CMJ was related with slower sprint and COD speed times (r = 0.54–0.87). However, these studies have been executed in youth male soccer players, and to the authors’ knowledge, no comparable data is available in adolescent female soccer players. Consequently, obtained results have not demonstrate conclusive findings when we try to determine the relation between inter-limb asymmetries and measures of physical performance, particularly in adolescent female soccer players.

Therefore, the objectives of this study were: (1) to measure inter-limb asymmetries in three distinct age groups in adolescent female soccer players, and (2) to determine the association between inter-limb asymmetries and measures of physical performance in different age groups.

## 2. Materials and Methods

### 2.1. Participants

Fifty-four adolescent female soccer players from three different teams of the same Spanish the club academy squad (Iberdrola Women’s First Division) participated in this study. They were distributed in three age groups: U-18 (*n* = 18; age: 16.9 ± 0.5 years; height 161.8 ± 9.2 cm; mass 57.7 ± 9.3 kg), U-16 (*n* = 21; age: 14.9 ± 0.5 years; height 159.8 ± 5.3 cm; mass 53.6 ± 8.1 kg) and U-14 (*n* = 15; age: 13.7 ± 0.6 years; height 154.1 ± 7.9 cm; mass 48.9 ± 7.9 kg). A priori power analysis identified that when aiming to assess differences between three independent groups at a statistical power of 0.8, with an alpha level of 0.05 and effect size of 0.8, 21 players were required for each group. Thus, the present study is under-powered. However, it is worth noting that the present group were adolescent female and studies using such samples are likely to be under-powered given the limited number of athletes associated with this specific population. All the players have more than 4 years of training experience in soccer. The physical training sessions, in all teams, consisted of training exercises for coordination, agility, speed and injury prevention that allow maintaining the level of physical condition. Participants were healthy and without any disease or injury that could interfere with the study results. Informed consents were obtained from all players involved in this investigation. In accordance with the Declaration of Helsinki, the informed consents of all the players were obtained and the study was approved by the Ethical Committee for Clinical Research of the Government of Aragon (CP19/039, CEICA, Zaragoza, Spain).

### 2.2. Procedures

To ensure the standardized distribution of the groups, all the players followed the same protocols during the two sessions of physical tests. Do not participate in any strenuous exercise 24 h in advance. Do not take the last meal 3 h before the tests. Don’t drink caffeinated beverages. The tests were performed at the same time of the day (6 p.m. to 8 p.m.). The first session was used to familiarize all participants with the jump, sprint and COD speed test. The second session, separated by 72 h, the tests were carried out in a random and balanced order, for the correct data collection. The order of the tests was jump, sprint and COD speed test. These tests were performed by the same group of investigators, and were carried out on days with stable environmental conditions measured by a wet bulb globe temperature monitor (~22 °C and ~20% humidity), days that did not comply with these environmental conditions were discarded, in an artificial grass soccer field where every team had their training sessions. All participants completed a rise, activate, mobilize and potentiate (RAMP) system warm-up protocol [20]. A 3-min rest period was provided after the last practice trial and the start of data collection. Three attempts per test were allowed with 3 min of passive recovery between repetitions. Players wore athletic shoes (for jump tests) and soccer boots (for linear sprint and COD test).

### 2.3. Single Leg Countermovement Jump Test 

To calculate the vertical jump capacity, a single leg CMJ was used the Optojump tool (Optojump, Microgate, Bolzano, Italy). All subjects were instructed to perform a maximum vertical jump with their hands on their hips and to land in a vertical position with their knees bent, controlled and balanced, and held in the landing position for 2–3 s. Three attempts were made and the best jump was selected for analysis.

### 2.4. Single Leg Hop Test 

To calculate single leg hop was used a standard measuring tape (30 m M13; Stanley, New Britain, CT, USA). Each subject started behind the starting line and jumped as far as possible (horizontal distance), landing on the same leg, controlled and balanced, and held in the landing position for 2–3 s [21]. Three attempts were made and the best jump was selected for analysis.

### 2.5. 40-m Sprint Test

To calculate the running speed, it was recorded with photoelectric cells (Microgate). The sprint time of 40 m, and the partial times of 10, 20 and 30 m were measured. Subjects like previous studies started with the front foot 0.5 m before the start [22]. The photoelectric cells were mounted on tripods 0.75 m above the ground and 3 m apart [23]. The test was prepared, and the player chose the moment of departure. The time began to count when the player cut the first photocell. Subjects were given verbal encouragement during each sprint. Three attempts were made and the best time was selected for analysis.

### 2.6. 180° Change of Direction Speed Test

To calculate the 10-m sprint test with a 180° COD, it was recorded with photoelectric cells (Microgate). The 180° COD is a modification of the 505 test [24], with good test-retest reliability [25]. Subjects started with the front foot 0.5 m before the start. and a test was carried out at a maximum speed of 10 m. plus 5 m, turn 180° on the right or left foot and 5 m to the finish line. The photoelectric cells were mounted on tripods 0.75 m above the ground and 3 m apart [23]. The test was prepared, and the player chose the moment of departure. The time began to count when the player cut the first photocell. Subjects were given verbal encouragement during each repetition. Three attempts were made and the best time was selected for analysis.

### 2.7. Statistical Analysis 

All data were recorded as mean and standard deviation (SD). Normality was analyzed with the Shapiro-Wilk test and none of the variables had a normal distribution. Within-session reliability of test measures was computed using a two-way random intraclass correlation coefficient (ICC) with absolute agreement and 95% confidence intervals, and the coefficient of variation (CV). The interpretation of the ICC values was excellent (>0.90), good (0.75–0.90), moderate (0.5–0.75) and bad (<0.50) [26] and as an acceptable criterion of responsibility a CV lower than 10% [27].

Noting that asymmetries may favour either side depending on which limb scores larger [4]. The consistency of the asymmetries was calculated with the Kappa coefficient and they were interpreted as poor (≤0), mild (0.01–0.20), regular (0.21–0.40), moderate (0.41–0.60), substantial (0.61–0, 80), almost perfect (0.81–0.99) and perfect [28]. SPSS statistical software (Version 19.0; SPSS Inc, Chicago, IL, USA) was used.

Inter-limb asymmetries were quantified as the percentage difference between the two limbs using the following equation [29].

100/Max value (right and left) * Min value (right and left) * −1 + 100

A one-way analysis of variance was conducted to determine systematic bias between age groups for mean test scores and asymmetry values, with statistical significance set at *p* < 0.05 identified via Bonferroni post-hoc analysis. The relationships between inter-limb asymmetry scores and test scores were analysed using Spearman’s ρ correlations. To determine the magnitude of differences between the groups for each variable, effect sizes (ES) were calculated using standardized mean difference corrected as Hedges’g [30]. These were interpreted in line with Hopkins et al. [31] where trivial (<0.2), small (>0.2–0.6), moderate (>0.6–1.2), large (>1.2–2.0), very large (>2.0–4.0) and near perfect (>4.0). 

## 3. Results

Table 1 shows the reliability within the session and shows high reliability except for COD of 180° (ICC: 0.85–0.87) and linear velocity of 10 m (ICC: 0.83), while acceptable CV was obtained for all tests (<10%). 

Mean test scores and ES for each group are presented in Table 2. The U-18 group performed significantly better jumps and faster times than the U-14 in single leg hop test left and 180° COD left. The U-16 group were significantly faster than the U-14 over 40-m and 180° COD left. No other significant differences between groups were found. 

When comparing ES, trivial to moderate differences were evident between all group comparisons. Table 3 shows the levels of agreement for the asymmetry scores (Kappa coefficient).

**Figure 1:**
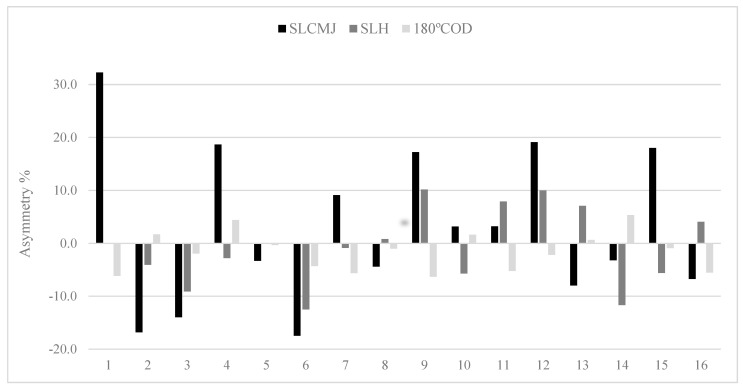
Data about individual asymmetry for single leg countermovement jump (SLCMJ), single leg hop (SLH) and 180° change of direction (180° COD) in the U-14 group. N.B: above 0 indicates right leg dominance and below 0 indicates left leg dominance.

**Figure 2:**
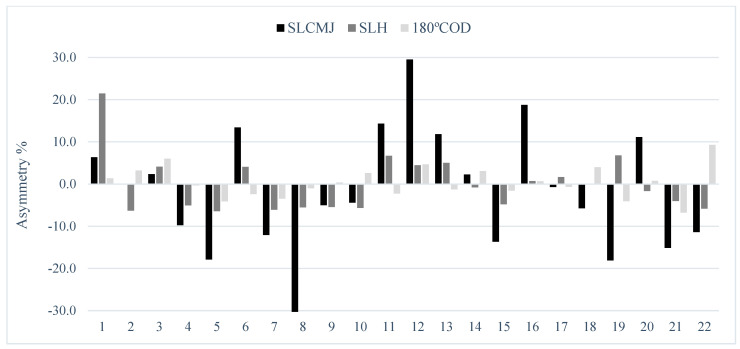
Data about individual asymmetry for single leg countermovement jump (SLCMJ), single leg hop (SLH) and 180° change of direction (180° COD) in the U-16 group. N.B: above 0 indicates right leg dominance and below 0 indicates left leg dominance.

**Figure 3:**
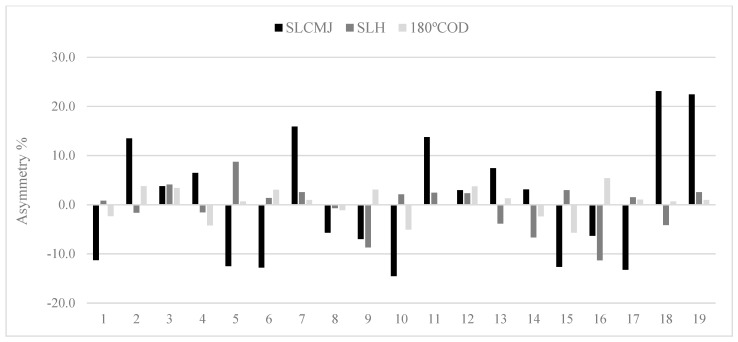
Data about individual asymmetry for single leg countermovement jump (SLCMJ), single leg hop (SLH) and 180° change of direction (180° COD) in the U-18 group. N.B: above 0 indicates right leg dominance and below 0 indicates left leg dominance.

The results showed substantial levels of agreement of U-16 between single leg CMJ and single leg hop test (0.45). The rest of the groups show poor to fair levels (range: −0.31 to 0.24) for all comparisons. Owing to the variable nature in both the magnitude and direction of asymmetry, individual inter-limb differences are presented for jump and COD speed tests in U-14 (Figure 1), U-16 (Figure 2) and U-18 (Figure 3) female soccer player. 

Table 4 shows Spearman’s *ρ* correlations between vertical and horizontal inter-limb asymmetry scores and tests data. No significant relationships were present between single leg CMJ and single leg hop inter-limb asymmetry scores and sprint or COD speed performance. Spearman’s *ρ* correlations between COD speed inter-limb asymmetry scores and tests are shown in Table 5. No significant relationships were found between COD speed inter-limb asymmetry scores and sprint or jump performance.

## 4. Discussion

The objectives of this study were to establish inter-limb asymmetry scores from the single leg CMJ, single leg hop test and 180° COD speed in adolescent female soccer players across different age groups, and to determine the relationship between these asymmetries and measures of physical performance. Outcomes demonstrated different magnitudes of asymmetry between tests. Vertical jump test showed larger asymmetry scores when comparing to horizontal jump test and COD speed test. There were no meaningful relationships between asymmetry scores and independent measures of physical performance. Moreover, asymmetries rarely favored the same side between jump and COD speed tests, highlighting the task-specific nature of inter-limb differences.

The totally of tests showed an exceptional relative reliability and an acceptable variability, and this can indicate that the results can be understood with confidence for next analysis [32]. The experience and the regular strength and conditioning training performed during the season may provide to the acceptable reliability of the data [33]. In relation to the asymmetry scores reported in the present study, the COD speed test (2.9%) showed a lower magnitude of asymmetry in comparison to the single leg CMJ test (11.6%), this is in contract with previous research [10,34]. Asymmetry is highly task-specific and although some tests produce larger asymmetry values, the inherent variability in asymmetry scores is still large for all tests, as shown by the varying magnitude of asymmetry at an individual level (see Figure 1, Figure 2 and Figure 3). For this reason, it is advisable that researchers, coaches and practitioners calculate not only result measures (e.g., jump height and reactive strength index) but also test variability [10]. This will enable practitioners to determine when asymmetries are ‘real’ (i.e., greater than the test error) or within the measured noise of the test [8]. 

An outstanding point to take into account from these results is that vertical jump (single leg CMJ: 11.6%) showed greater asymmetries than horizontal jump (single leg hop test: 4.8%), and this is in agreement with previous research [6,35]. In the same line, Bishop et al. [6] showed asymmetries of 12.5% and 6.8% in single leg CMJ and single leg hop test respectively, in elite youth female soccer players. It is possible that vertical jump may be more sensitive at identifying asymmetries because in these cases the study population are adolescent players. Children exercise horizontal hopping activities (e.g., hopscotch and somersault) from an early age [36,37]. These horizontal movement patterns are performed more than unilateral vertical tasks [6]. This can clarify the reason why inter-limb differences are not as horizontal jump, notwithstanding, more researches are still necessary to completely support this theory. 

No significant differences between groups were during the asymmetry of CMJ, single leg hop and COD tests (Table 2). Intuitively, given the physical maturity of older athletes in comparison with younger, it seems logical to assume that these players would be able to be better asymmetry scores. In addition, there is a tendency for females’ athletic performance to reach a plateau around the age of 13 years (puberty) [38], for this reason may be better asymmetry parameters in the older female groups in our study. This is supported in previous research by several studies which showed that older players outperformed younger players on jump and COD speed asymmetries [6,13,39,40,41]. 

The Kappa coefficient (Table 3) was calculated for the purpose of determining how usually asymmetries favoured the same side between tests. Results showed poor to fair levels of agreement for the side consistency of asymmetry between jumps and COD speed (Kappa range = −0.31 to 0.45). Simply put, if an asymmetry was favoured on the left limb during one of the jump tests, it was unlikely that the same side performed superiorly during the COD speed test. Previous research showed very low levels of agreement (<0.2) between different tests, indicating that asymmetries for these metrics favoured the imbalance [4,42]. In contrast, a comparison between both jumps in U-16 players showed moderate levels of agreement (Kappa: 0.45), corroborating that these asymmetries were more frequently present on the same side. The noticeably better levels of agreement between single leg CMJ and single leg hop test shows that these two tests shared some similarities in limb dominance, regardless of whether the focus was maximal jump height or distance. These results are partly in accordance with Bishop et al. [39] who showed a substantial level of agreement (Kappa: 0.61) between the squat jump and CMJ, in youth female soccer players. Consequently, these results demonstrate the variable nature of magnitude and direction of asymmetry and emphasize the require for more individual approach to data analysis [4] (Figure 1, Figure 2 and Figure 3).

In Table 1 we have observed that inter-limb asymmetry metrics vary depend on the test used, and for this reason it is evident that not all female players react similarly to the same test referring to asymmetry. In this regard, group mean asymmetry scores ranged from 2.91 to 11.6%; however, many individual asymmetry values exceeded these percentages, mainly in vertical jump test (Figure 1, Figure 2 and Figure 3). Literature has shown that asymmetries of >10% may decrease jump height [43] and increase COD speed times [44] indicating that the reduction of these differences may be favourable. Moreover, the direction of asymmetry to favour the left limb was observed in our results (i.e., non-dominant kicking limb) [10], in this case, 31–68% female players presented asymmetry on the left side for jump and COD speed tests. For these reasons, the individual information seems to be important to design a precise training program, with a view to reduce inter-limb asymmetries and therefore, to improve athletic performance and to decrease potential risk of injury [8,45]. In addition, other interesting methods for evaluating asymmetries could be used such us the Functional Movement Screen, ankle dorsiflexion or Y Balance test [40,46]. However, the problem to evaluate asymmetry from these tests, is that it is subjective interpretation of human movement quality [47]. Asymmetry is already a very noisy and variable concept, so to include subjective data could add more error to the equation.

There are no relevant relation between asymmetry results, emphasizing the independent nature of jumping, sprinting and COD speed test in adolescent female soccer players (Table 3 and Table 4). This finding is supported by preceding studies [10,11], which do not observe any correlation between different asymmetry results in female soccer players. Moreover, Bishop et al. [48] observed in a recent literature that comparing asymmetry scores over multiple tests levels of agreement were often poor (i.e., Kappa Coefficients < 0). Put simply, this indicates that the direction of asymmetry is rarely the same between tasks and provides further evidence of the task-specific nature of measuring side-to-side differences [49]. This highlights that if profiling asymmetry is deemed necessary, practitioners should do so using a variety of tests and not expect the same outcome between them. Furthermore, despite previous research in a comparable sample also showing no significant associations between asymmetry and independent measures of performance [11], the variable nature of asymmetry is undoubtedly a key factor in the lack of significant relationships with independent measures of athletic performance. 

In this regard, it has been suggested to locate possible bilateral differences and imbalances between limbs is necessary to performed more than one type of exercise [42,50]. In addition, inter-limb asymmetries can also influence performance (e.g., greater symmetrical team-sports players look like they are faster than their asymmetrical counterparts) [35]. In this respect, strength and plyometric training are two of the most often used strategies to enhance soccer performance and high-intensity actions, just like decreasing asymmetries [40,41,51]. Therefore, adolescent female soccer players should make strength and plyometric exercises to improve performance and also, to reduce asymmetries. 

In spite of the utility of these findings, the present study has several restrictions which must be recognized. Adolescent female soccer players have specific characteristics (e.g., anthropometry) and, so our outcomes cannot be extrapolated to other sports. Information on participation in other sports was not collected in the current sample. There were no differences in playing position due to the limited number of players presented in every single playing role. Consequently, we strongly recommend that future studies be conducted with the wider statistical population, collecting information about participation in other sports and considering more players for each playing positions. In addition, practitioners working in soccer may wish to consider defining limbs as ‘dominant’ and ‘non-dominant’, in respect to the preferred kicking limb; not as right and left, as in the present study. However, it is worth noting that this method does not always guarantee that the dominant limb will be the superior performing limb [10,52]. With that in mind, if limbs are defined within the context of dominance, close attention to the raw test scores (not just asymmetry) is required, so that the weaker or under-performing limb can be accurately identified. With this information, practitioners can accurately determine whether targeted training interventions are required. 

## 5. Conclusions

In conclusion, jumping and COD physical tests show asymmetries in adolescent female soccer players, but these asymmetries do not interfere with their physical performance. The largest asymmetry was observed in the single leg countermovement jump, and no asymmetries between groups (U-18, U-16 and U-14) were found. Finally, the direction of asymmetry appears highly variable, so the individual analysis of asymmetries should be consider to perform more precise training interventions on an individual level.

## Figures and Tables

**Table 1:** Mean test scores ± standard deviation (SD) and reliability for all players.

Test	Mean ± SD	% Asymmetry	CV (%)	ICC (95% CI)
SLCMJ_R_ (cm)	11.4 ± 2.79	11.6 ± 7.54	4.3	0.95 (0.88; 0.98)
SLCMJ_L_ (cm)	11.3 ± 3.05		5.8	0.95 (0.87; 0.98)
SLH_R_ (cm)	120.9 ± 15.9	4.82 ± 3.87	2.8	0.96 (0.89; 0.98)
SLH_L_ (cm)	121.1 ± 12.6		2	0.93 (0.82; 0.97)
180° COD_R_ (s)	3 ± 0.14	2.91 ± 2.10	1.4	0.87 (0.69; 0.95)
180° COD_L_ (s)	3.01 ± 0.16		1.4	0.85 (0.65; 0.94)
10 m (s)	2.08 ± 0.10	-	1.8	0.83 (0.62; 0.93)
20 m (s)	3.61 ± 0.17	-	1.1	0.94 (0.84; 0.97)
30 m (s)	5.10 ± 0.24	-	0.6	0.98 (0.95; 0.99)
40 m (s)	6.64 ± 0.34	-	0.6	0.98 (0.96; 0.99)

Note: SLCMJ: Single leg countermovement jump; SLH: Single leg hop test; 180° COD: 5 + 5 sprint test with a 180° change of direction; L: Left; R: Right; CV: Coefficient of variation; ICC: Intraclass correlation coefficient; CI: Confidence intervals.

**Table 2:** Mean data ± standard deviation for each age group and effect sizes between groups.

Test	U-18	U-16	U-14	ES U-18 vs. U-16	ES U-18 vs. U-14	ES U-16 vs. U-14
SLCMJ_R_ (cm)	11.9 ± 2.6	11.7 ± 3.08	10.4 ± 2.34	−0.07 (trivial)	−0.57 (small)	−0.51(small)
SLCMJ_L_ (cm)	11.9 ± 3.2	11.8 ± 2.86	10.1 ± 2.77	0.02 (trivial)	−0.66 (moderate)	−0.76 (moderate)
As CMJ (%)	10.9 ± 5.96	11.6 ± 8.47	12.1 ± 8.34	0.09 (trivial)	0.16 (trivial)	0.05 (trivial)
SLH_R_ (cm)	123.1 ± 12.6	123.6 ± 18.6	113.7 ± 10.2	0.11(trivial)	−0.63 (moderate)	−0.74 (moderate)
SLH_L_ (cm)	124.1 ± 3.6 *	123.1 ±13.5	114.5 ± 8.95	0.04 (trivial)	−0.72 (moderate)	−0.90 (moderate)
As SLH (%)	3.68 ± 3.01	5.11 ± 4.19	5.77 ± 4.19	0.04 (trivial)	0.57 (small)	0.15 (trivial)
180° COD_R_ (s)	2.96 ± 0.14	2.99 ± 0.14	3.07 ± 0.13	−0.03 (trivial)	0.40 (small)	0.90 (moderate)
180° COD_L_ (s)	2.95 ± 0.14 *	2.98 ± 0.13 **	3.12 ± 0.15	−0.01(trivial)	0.80 (moderate)	0.74 (moderate)
As COD (%)	2.57 ± 1.77	2.91 ± 2.31	3.32 ± 2.20	0.16 (trivial)	0.37 (small)	0.17 (trivial)
10 m (s)	2.05 ± 0.10	2.08 ± 0.10	2.12 ± 0.10	−0.29 (small)	0.23 (small)	0.69 (moderate)
20 m (s)	3.57 ± 0.17	3.63 ± 0.17	3.66 ± 0.14	−0.32 (small)	0.72 (moderate)	1.13 (moderate)
30 m (s)	5.04 ± 0.25	5.12 ± 025	5.16 ± 0.20	−0.32 (small)	0.60 (moderate)	0.48 (small)
40 m (s)	6.53 ±0.34	6.62 ± 0.34 **	6.84 ± 0.26	−0.27 (small)	1.03 (moderate)	0.87 (moderate)

Note: SLCMJ: Single leg countermovement jump; SLH: Single leg hop test; 180° COD: 5 + 5 sprint test with a 180° change of direction; L: Left; R: Right; ES: Effect size. U-18: Under 18; U-16: Under 16; U-14: Under 14. * Significant difference (*p* < 0.05) between U-18 and U-14 players. ** Significant difference (*p* < 0.05) between U-16 and U-14 players.

**Table 3:** Kappa coefficients and descriptive levels of concordance of asymmetries between the jumping speed and COD tests.

Test Comparison	Kappa Coefficient	Descriptor
Under-14:		
SLCMJ- SLH	0.13	Slight
SLCMJ-180° COD	−0.13	Poor
SLH-180° COD	−0.31	Poor
Under-16:		
SLCMJ- SLH	0.45	Moderate
SLCMJ-180° COD	−0.03	Poor
SLH-180° COD	−0.03	Poor
Under-18:		
SLCMJ- SLH	−0.16	Poor
SLCMJ-180° COD	0.24	Fair
SLH-180° COD	0.10	Fair

Note: SLCMJ: Single leg countermovement jump; SLH: Single leg hop test; 180° COD: 5 + 5 sprint test with a 180° change of direction.

**Table 4:** Spearman’s *ρ* correlation between vertical and horizontal jump height asymmetry and test across age groups.

Test	Asymmetry SLCMJ			Asymmetry SLH		
	U-14	U-16	U-18	U-14	U-16	U-18
180° COD _R_ (s)	−0.11	−0.16	−0.26	0.44	−0.06	0.50
180° COD _L_ (s)	0.11	0.06	−0.36	0.35	−0.01	0.25
10 m (s)	−0.25	−0.05	−0.06	−0.06	−0.32	0.25
20 m (s)	−0.17	0.05	0.00	0.07	−0.34	0.25
30 m (s)	−0.21	0.07	0.09	0.04	−0.33	0.26
40 m (s)	0.10	0.10	0.12	−0.06	−0.33	0.22

Note: SLCMJ: Single leg countermovement jump; SLH: Single leg hop test; 180° COD: 5 + 5 sprint test with a 180° change of direction; L: Left; R: Right; U-18: Under 18; U-16: Under 16; U-14: Under 14.

**Table 5:** Spearman’s *ρ* correlation between change of direction (COD) speed asymmetry and test across age groups.

Test	Asymmetry COD Speed		
	U-14	U-16	U-18
SLCMJ_R_ (cm)	−0.01	−0.27	−0.32
SLCMJ_L_ (cm)	−0.01	−0.01	−0.14
SLH_R_ (cm)	−0.16	−0.49	−0.16
SLH_L_ (cm)	−0.48	−0.39	−0.09
10 m (s)	0.48	0.24	0.35
20 m (s)	0.51	0.25	0.25
30 m (s)	0.45	0.26	0.16
40 m (s)	0.49	0.26	0.12

Note: SLCMJ: Single leg countermovement jump; SLH: Single leg hop test; 180° COD: 5 + 5 sprint test with a 180° change of direction; L: Left; R: Right; U-18: Under 18; U-16: Under 16; U-14: Under 14.

## Data Availability

The datasets generated and analyzed for this study can be requested by correspondence authors in epardos@usj.es and dlozano@usj.es.
